# The predictive value of dynamic monitoring of peripheral blood lymphocyte to monocyte ratio in patients with extranodal NK/T cell lymphoma

**DOI:** 10.1186/s12935-019-0993-9

**Published:** 2019-10-22

**Authors:** Shengnan Zhang, Mengjuan Li, Fangfang Yuan, Lin Chen, Ruihua Mi, Xudong Wei, Yongping Song, Qingsong Yin

**Affiliations:** Department of Hematopathy, Henan Institute of Hematology, Cancer Hospital Affiliated to Zhengzhou University, Zhengzhou, 450000 Henan China

**Keywords:** Extranodal NK/T cell lymphoma, Ratio of lymphocyte to monocyte, Dynamic monitoring, Prognosis

## Abstract

**Background:**

To investigate the value of dynamic monitoring peripheral blood lymphocyte-to-monocyte (LMR) ratio in evaluating the treatment response and prognosis of patients with extranodal NK/T cell lymphoma (ENKTL).

**Methods:**

A total of 148 patients with ENKTL were retrospectively analyzed in the Affiliated Tumor Hospital of Zhengzhou University between March 2012 and March 2018. The optimal cut-off value of LMR was determined using the receiver operating characteristic curve (ROC) method, then patients were divided into low LMR group and high LMR group. The LMR level was dynamically measured at various time points, and the relationships between LMR and therapeutic response, and survival were analyzed.

**Results:**

The complete remission rate (CR) was 85.7% in patients with high LMR at diagnosis, which was remarkably higher than that of patients with low LMR at diagnosis (64.9%) (*P *= 0.009). The 5-year overall survival (OS) and progression-free survival (PFS) were 49.28% and 44.89% in the low LMR group, respectively; 5-year OS and PFS in the high LMR group were 84.50% and 67.12%, respectively, significantly longer (*P* values were < 0.001 and 0.034, respectively). The OS and PFS of patients with elevated LMR after treatment were longer than those with decreased LMR after treatment (all *P* values < 0.05). The LMRs at relapse were significantly lower in both high and low LMR groups than those of the last follow-up (*P* values were 0.001 and 0.016, respectively). Univariate and multivariate analysis demonstrated that low LMR was an independent risk factor for poor prognosis in ENKTL patients (*P* values were < 0.001 and 0.009, respectively).

**Conclusions:**

Lymphocyte to monocyte ratio can be used as an indicator of treatment response, prognosis and recurrence in patients with ENKTL. Low LMR before and after treatment is a poor prognostic factor.

## Background

Extranodal NK/T cell lymphoma (ENKTL) is a unique histopathologic subtype of non-Hodgkin’s lymphoma (NHL), characterized by vascular damage and destruction, necrosis, cytotoxic phenotype, and Epstein–Barr virus (EBV) infection [1]. ENKTL, rare in Europe and North America, is more prevalent in Asia and South America, making up about 5–16% of all NHL patients in China [2]. ENKTL is a highly heterogeneous malignant lymphoma. Almost half of patients have a rapid disease progression or recurrence. Once ENKTL becomes resistant to chemotherapy, there is no standard and effective treatment, and the prognosis is poor [3]. The early identification of high-risk patients is essential for clinical decision-making. Therefore, several studies have tried to identify new prognostic markers.

Serum lactate dehydrogenase (LDH) reflects cell proliferation activity, and the elevated LDH is a parameter reflecting poor prognosis. The prognostic value of LDH has been confirmed in non-Hodgkin’s lymphoma (NHL), and included in the International Prognostic Index (IPI) [4]. Besides, chronic inflammation has been demonstrated to be related to tumor development and progression [5]. Proinflammatory chemokines and cytokines in tumor microenvironments contribute to the proliferation and survival of malignant cells [6, 7]. Inflammatory indexes such as c-reactive protein (CRP), lymphocyte, and monocyte have been reported to have prognostic values in patients with ENKTL [8–10]. Lymphocyte is an important biomarker which reflects the host’s immune status, and lymphopenia is associated with a poorer survival in various subtypes of NHL, including ENKTL. Monocyte is thought to be surrogate markers of tumor microenvironments [8, 9]. These findings suggest that lymphocytes and monocytes are involved in anti-tumor immune response as well as formation of the tumor microenvironments. Thus, the lymphocyte to monocyte ratio (LMR) has potential prognostic value in other malignant diseases [11–13]. Nevertheless, the value of LMR in ENKTL patients has not been fully characterized.

Therefore, the purpose of this study was to investigate the predictive value of dynamic monitoring of LMR in peripheral blood at various time points before and after therapy in patients with ENKTL.

## Patients and methods

### Patients

This study was a retrospective analysis of a cohort of 148 newly-diagnosed ENKTL patients treated in the Affiliated Tumor Hospital of Zhengzhou University between March 2012 to March 2018. The inclusion criteria are as follows: (1) pathologically and immunohistochemically confirmed diagnosis of ENKTL according to the WHO 2016 classification of the tumors and hematopoietic and lymphoid tissues [14], and was validated by more than two experienced professors from the Department of Pathology; (2) sufficient data during treatment and follow-up. Patients would be excluded from this study if they had a history of anti-tumor treatment, and clinical evidence of acute infection or chronic acute inflammatory disease.

Data collected included Eastern Cooperative Oncology Group (ECOG), performance status (PS), age, gender, involved sites, B symptoms, serum LDH level, serum β2 microglobulin (β2 M), bone marrow involvement, complete blood count (CBC), absolute lymphocyte count (ALC), absolute monocyte count (AMC), liver and renal function, serum potassium, sodium, chloride and calcium levels, International Prognostic Index (IPI), Korean Prognosis Index (KPI) indices, and stage (Lugano staging system). This study was approved by the Medical Ethical Committee of the Affiliated Tumor Hospital of Zhengzhou University. All data of the recruited patients were obtained after informed consent in accordance with the Declaration of Helsinki.

### The measurement of the absolute lymphocyte/monocyte count and the calculation of LMR and LMR/LDH

Complete blood count and serum LDH level were measured and recorded at various time points (at diagnosis, before every other course of chemotherapy, and during follow-up). The LMR was calculated through ALC divided by AMC. Similarly, the LMR/LDH was calculated through LMR value divided by serum LDH value. Namely, the LMR and LMR/LDH at diagnosis were obtained from data within a week before the first course of chemotherapy; the LMR during treatment was from data before every other course of chemotherapy; the LMR during follow-up was from data every 3 months in the 1st year, every 6 months in the 2nd year, and then once yearly. The optimal cut-off values of both LMR and LMR/LDH were determined using Cutoff Finder via the receiver operating characteristic curve (ROC) method (Euclidean distance).

### Regimens and responses

Patients with early stage disease treated with 4–6 cycles of chemotherapy followed by involved-field radiotherapy (IFRT). Patients with advanced disease treated with 6–8 cycles of chemotherapy as first-line treatment; subsequently, IFRT could be delivered as consolidation, palliative, or salvage therapy depending on physician condition of patients. Of the 148 patients, 5 (3.38%) received radiotherapy alone, and 143 (96.62%) received chemotherapy, of which 94 (63.5%) received chemotherapy plus radiotherapy, and 39 (26.4%) received chemotherapy alone. The chemotherapy regimens were shown in Table 1. The proportions of patients treated with each regimen maintained a balance between the high and low LMR groups.

**Table 1:** Clinical characteristics of 148 patients by the LMR level at diagnosis

Characteristic	No. (%)	Low LMR group LMR ≤ 2.7	High LMR group LMR > 2.7
Gender			
Male	91 (61.5)	22 (14.9)	69 (46.6)
Female	57 (38.5)	15 (10.1)	42 (28.4)
Age (year)			
> 60	18 (12.2)	6 (4.1)	12 (8.1)
≤ 60	130 (87.8)	31 (20.9)	99 (66.9)
B symptoms			
Absent	65 (43.9)	14 (9.5)	51 (34.5)
Present	83 (56.1)	23 (15.5)	60 (40.5)
Lugano stage			
I–II_2_	119 (80.4)	22 (14.9)	97 (65.5)
III–IV	29 (19.6)	15 (10.1)	14 (9.5)
ECOG PS			
< 2	143 (96.6)	35 (23.6)	108 (73.0)
≥ 2	5 (3.4)	2 (1.4)	3 (2.0)
LDH			
Normal	107 (72.3)	23 (15.5)	84 (56.8)
Abnormal	41 (27.7)	14 (9.5)	27 (18.2)
IPI score			
0–2	137 (92.6)	32 (21.6)	105 (70.9)
3–5	11 (7.4)	5 (3.4)	6 (4.1)
Distant metastasis			
Yes	97 (65.5)	22 (14.9)	65 (43.9)
No	51 (35.5)	15 (10.1)	36 (24.3)
Chemotherapy regimen			
DIE-L	59 (39.9)	45 (30.4)	14 (9.5)
GDP-L	37 (25.0)	28 (18.9)	9 (6.1)
CHOP-L	22 (14.9)	17 (11.5)	5 (3.4)
SMILE	19 (12.8)	13 (8.8)	6 (4.1)
P-Gemox	6 (4.1)	4 (2.7)	2 (1.4)

*DIE-L* ifosfamide, etoposide, dexamethasone, and l-asparaginase, *GDP-L* gemcitabine, dexamethasone, cisplatin, and l-asparaginase, *CHOP-L* cyclophosphamide, doxorubicin, vincristine, prednisolone, and l-asparaginase, *SMILE* dexamethasone, methotrexate, ifosfamide, l-asparaginase, and etoposide, *P-Gemox* gemcitabine, oxaliplatin, and pegaspargase

Overall survival (OS) is defined as the duration from disease diagnosis to the last follow-up or death. Progression-free survival (PFS) is defined as the duration from disease diagnosis to disease progression or disease-related death. These treatment responses were evaluated every two courses of chemotherapy through clinical manifestations, B-ultrasound, Computed Tomography (CT), Magnetic Resonance Imaging (MRI), or Positron Emission Computed Tomography (PET-CT). According to standard response criteria for NHL [15], the responses were divided into complete remission (CR), CRu (CR, unidentified), partial remission (PR), stable disease (SD), and progressive disease (PD), and (CR + CRu + PR) served as overall response rate (ORR).

In the follow-up analysis, CBC, blood chemistries (including liver and renal function, LDH, β2 M, etc.) and imaging (including B-ultrasound, CT, MRI, or PET-CT) were performed every 3 months in the 1st year, every 6 months in the 2nd year, and then once yearly. Morphological examination of bone marrow cells was performed in patients with bone marrow infiltration at the beginning of disease. The follow-up deadline was September 2018. The survival time of lost patients was calculated to the last follow-up date.

### Statistical analysis

Categorical characteristics and ratio were compared using the Chi-square or Fisher’s exact test. The Kaplan–Meier method was used to calculate the probability of survival, and difference between groups were compared by log-rank test. Cox proportional-hazards regression model was applied to perform Univariate and multivariate. A two-sided *P* < 0.05 was defined as significance. All statistical analyses were conducted using SPSS 21.0 and Graphpad Prism 7.0 software.

## Results

### Patient characteristics

A total of 148 cases of newly diagnosed ENKTL patients, with median age was 42 years (range 11–72 years), were eligible to be evaluated. 37 patients (25.0%) experienced recurrence or disease progression, and 24 patients (16.2%) died. The median follow-up time was 32 months (range 6–74 months). The majority of patients were < 60 years, Lugano stage I/II_2_ was dominant. Among 148 patients, 42 (28.4%) were positive for serum EBV-DNA, 83 (56.1%) were negative for serum EBV-DNA, and 23 (15.5%) were undetected. The median ALCs were 1.4 × 10^9^/L (range 0.23–3.35), and the median AMCs were 0.35 × 10^9^/L (range 0.02–0.84). 148 eligible patients were categorized into the high and the low LMR groups according to the optimal cut-off value of the LMR or LMR/LDH, respectively. The characteristics were shown in Table 1.

### The optimal cut-off values of the LMR and LMR/LDH

**Fig. 1:**
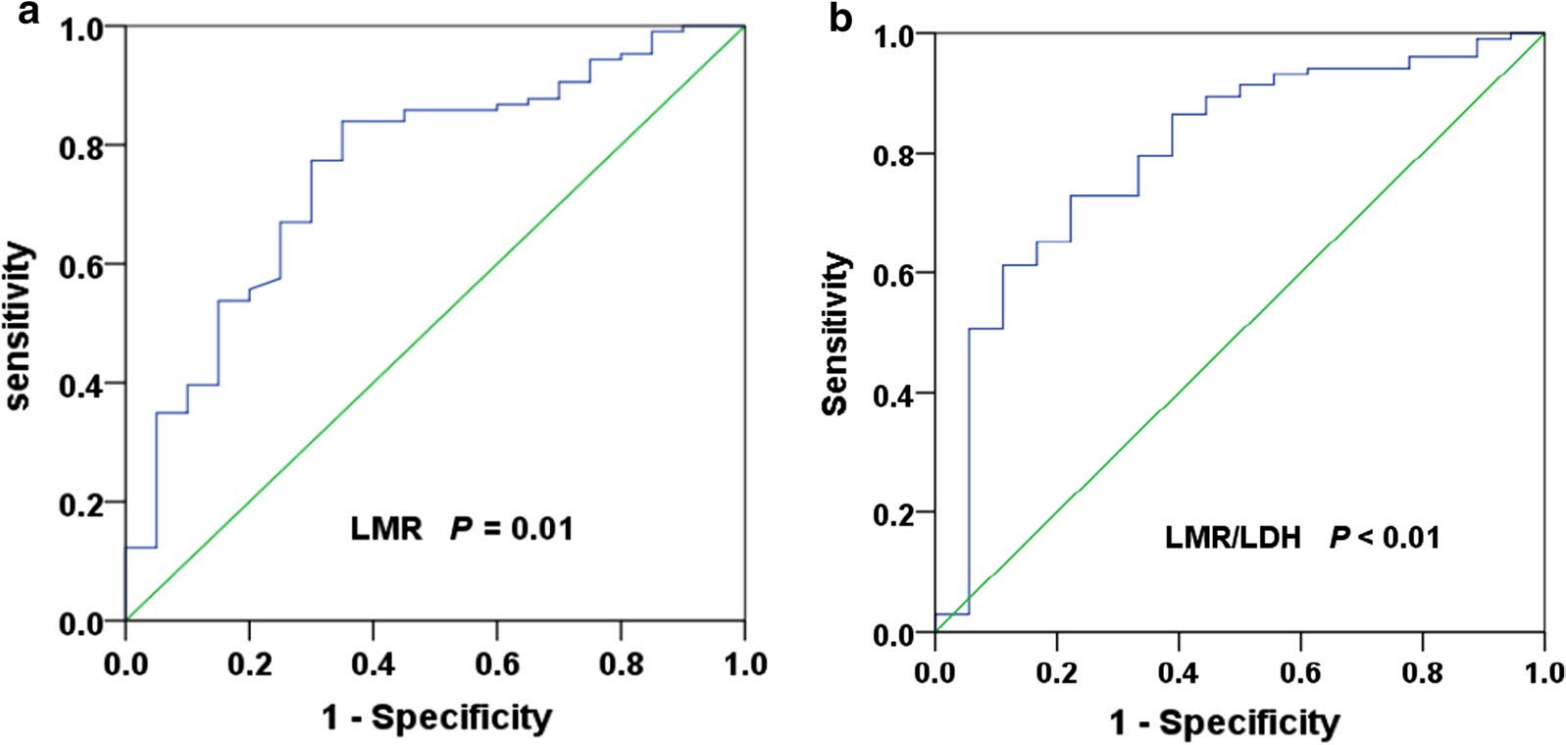
ROC curve analysis of the LMR and LMR/LDH ratio at diagnosis. **a** The optimal cut-off value of LMR was 2.70 (AUC was 0.738, 95% CI 0.619–0.856, *P* = 0.01). **b** The optimal cut-off value of LMR/LDH ratio was 1.07 (AUC was 0.758, 95% CI 0.618–0.898, *P* < 0.01)

The optimal cut-off value of LMR was 2.70, and the area under curve (AUC) was 0.738 (95% CI 0.619–0.856), *P* = 0.01 (Fig. 1a). A total of 111 patients (75.0%) with LMR > 2.70 at diagnosis were defined as the high LMR group, and 37 patients (25.0%) with LMR ≤ 2.70 at diagnosis were defined as the low LMR group. Similarly, the optimal cut-off value of LMR/LDH ratio was 1.07, and the area under curve (AUC) was 0.758 (95% CI 0.618–0.898), *P* < 0.01 (Fig. 1b). A total of 103 patients (69.6%) had LMR/LDH ≥ 1.07, and 45 patients (30.4%) had LMR/LDH < 1.07.

### LMR at diagnosis predicted the early response to treatments and prognosis in ENKTL patients

The CR rate in the high LMR group at diagnosis (85.7%) was remarkably higher than that in the low LMR group (64.9%) (*P* = 0.009). The LMR significantly reduced when the high LMR group reached the good response (CR + PR) (P = 0.032); however, there was no statistical change among the non-response patients (P = 0.098). There was no statistical change in the low LMR group regardless of whether the response was achieved or not (*P*-values were 0.074 and 0.264, respectively).

**Fig. 2:**
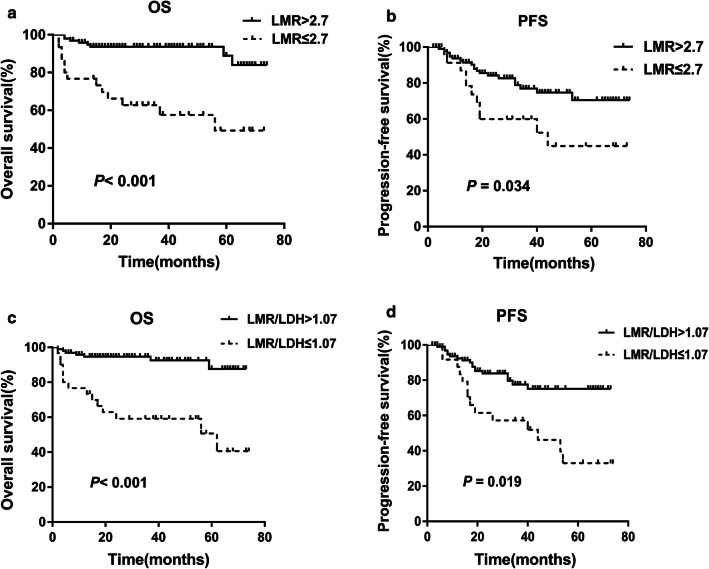
The LMR and LMR/LDH ratio at diagnosis can predict clinical outcomes in patients with ENKTL. **a**, **b** The low LMR at diagnosis were significantly related to poorer OS and PFS (*P* value were < 0.001 and 0.034, respectively). **c**, **d** The low LMR/LDH at diagnosis were significantly associated with shorter OS and PFS (*P* value were < 0.001 and 0.019, respectively)

The OS (Fig. 2a, *P *< 0.001) and PFS (Fig. 2b, *P *= 0.034) in the low LMR group at diagnosis were significantly lower than those in the high LMR group. The OS (Fig. 2c, *P *< 0.001) and PFS (Fig. 2d, *P *= 0.019) in the low LMR/LDH group at diagnosis were significantly lower than those in the high LMR/LDH group.

### The survival rate of patients with increased LMR after treatment was greater than that of patients with decreased LMR after treatment

**Fig. 3:**
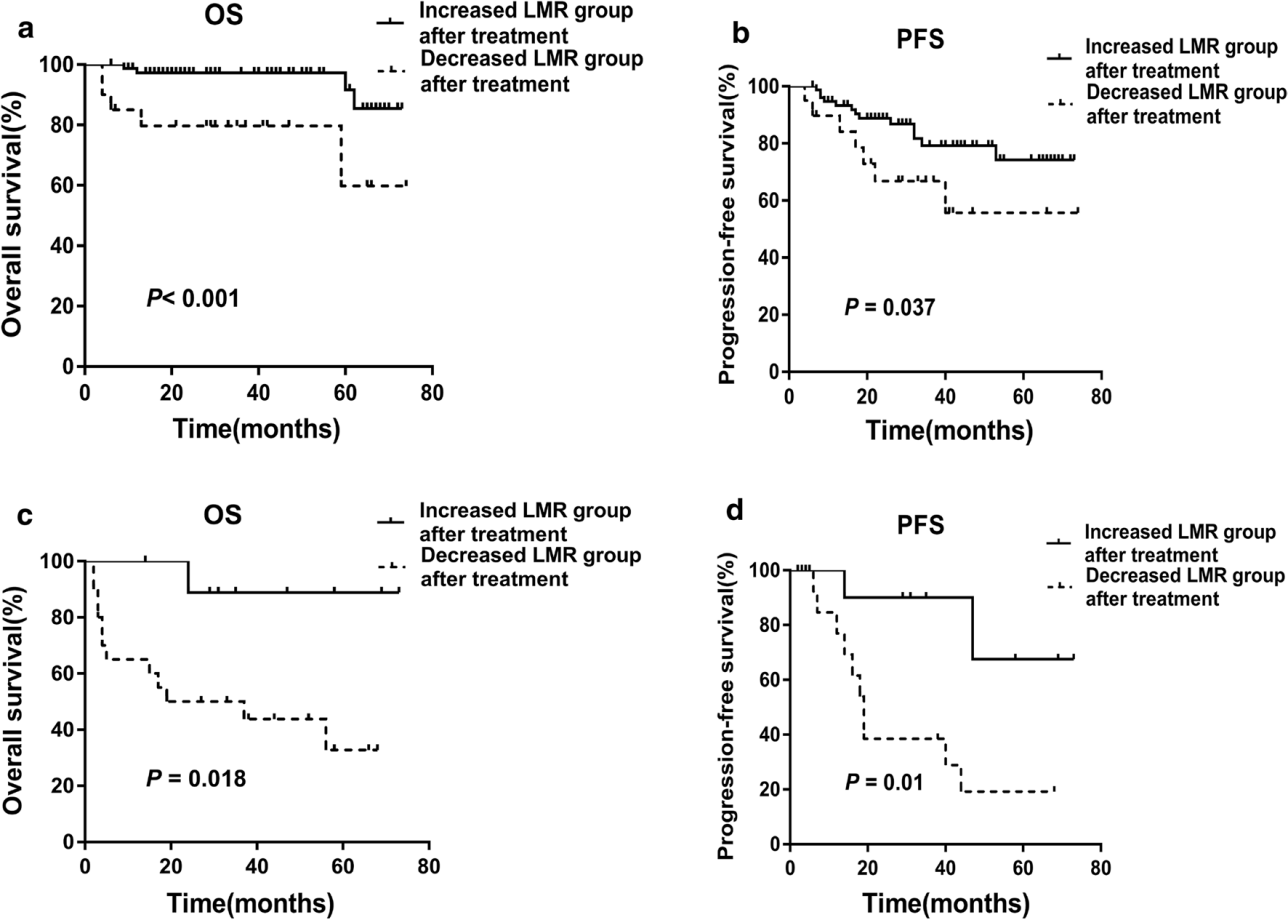
The OS and PFS in patients with increased LMR after treatment were better than that in patients with decreased LMR after treatment. **a**, **b** The OS and PFS in patients with increased LMR after treatment were better than that of patients with decreased LMR after treatment in the high LMR group at diagnosis (*P* value were < 0.001 and 0.037, respectively). **c**, **d** The OS and PFS in patients with increased LMR after treatment were better than that of patients with decreased LMR after treatment in the low LMR group at diagnosis (*P* value were 0.018 and 0.01, respectively)

According to the change of LMR level during treatment, the patients with high LMR pretreatment were stratified into two subgroups: Group 1, those with increased LMR at the end of therapy (≥ the LMR level at diagnosis) (n = 80), and Group 2, those with no increase in LMR after treatment (< the LMR level at diagnosis) (n = 24). Patients in Group 1 had longer OS (Fig. 3a, *P* < 0.001) and PFS (Fig. 3b, *P *= 0.037) than those in Group 2. Patients with low LMR pretreatment were stratified into two subgroups: Group 3, those with increased LMR at the end of therapy (≥ the LMR level at diagnosis) (n = 15), and Group 4, those with no increase in LMR (< the LMR level at diagnosis) (n = 17). Similarly, patients in Group 3 had longer OS (Fig. 3c, *P *= 0.018) and PFS (Fig. 3d, *P* = 0.01) than those in Group 4.

**Fig. 4:**
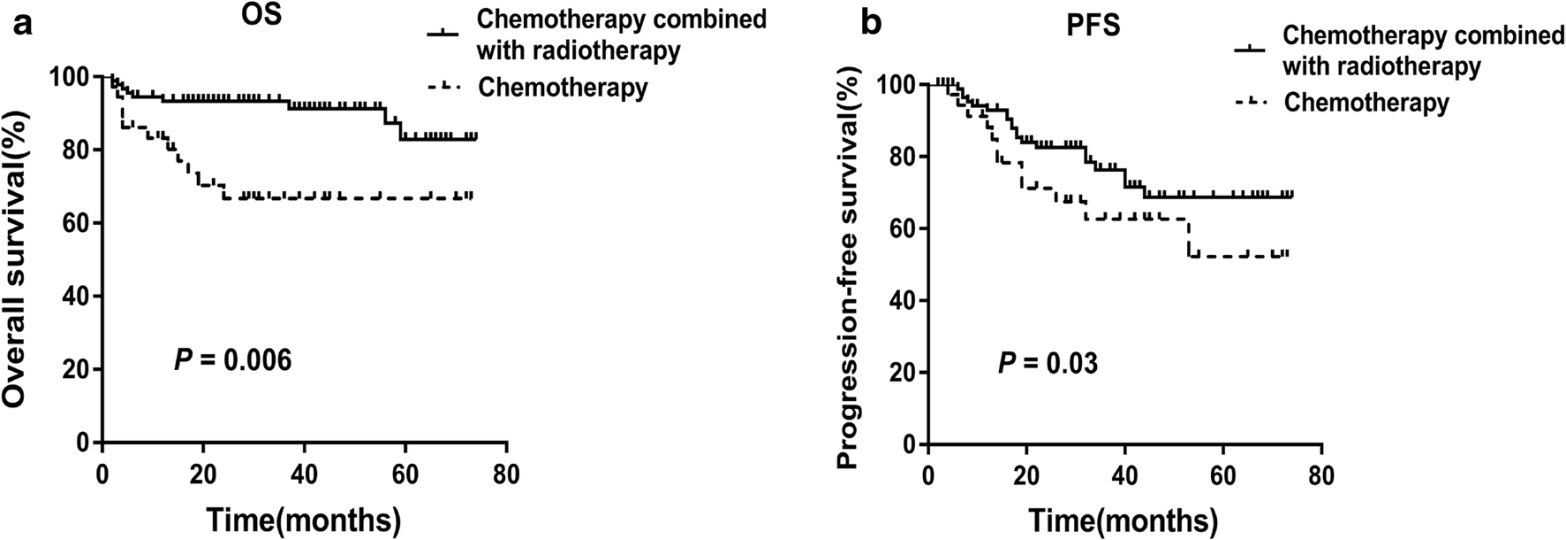
Chemotherapy combined with radiotherapy improved the survival of patients with ENKTL. **a**, **b** The OS and PFS of patients received chemotherapy plus radiotherapy were significantly longer than those of patients received chemotherapy alone (*P* value were 0.006 and 0.03, respectively)

**Fig. 5:**
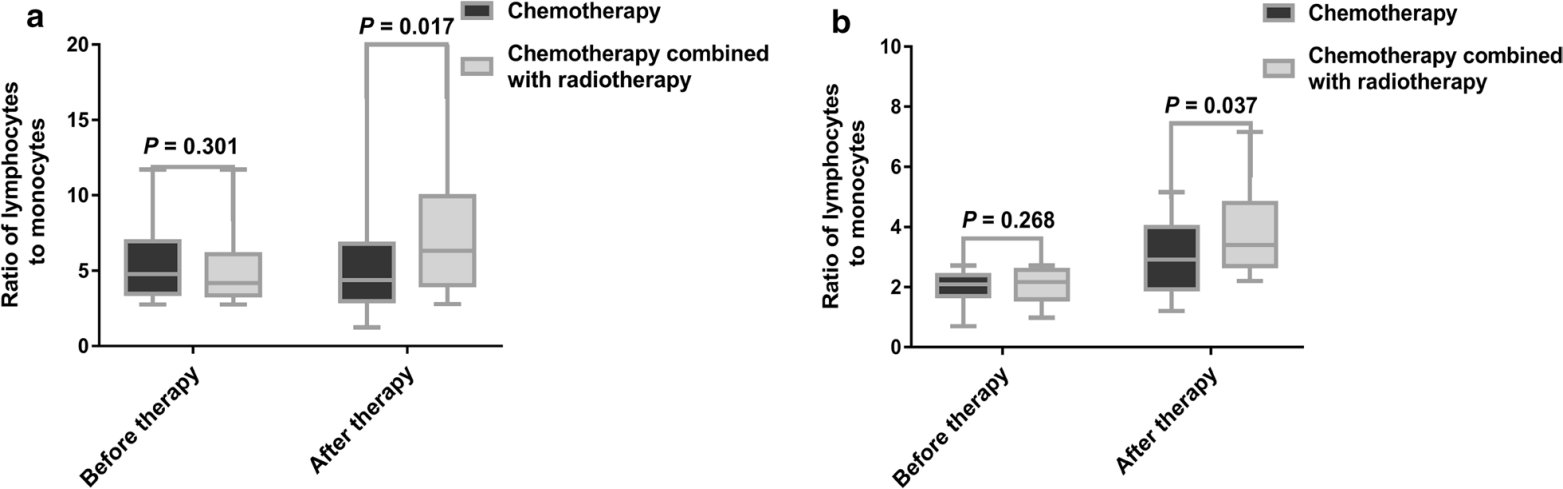
Chemotherapy plus radiotherapy regimen promotes the increase of LMR levels at the end of treatment in both groups with high and low LMR level at diagnosis. **a**, **b** The LMR of patients received chemotherapy plus radiotherapy was higher than that of patients received chemotherapy alone at the end of treatment in both high and low LMR groups (*P* value were 0.017 and 0.037, respectively)

There was no statistical difference in survival among patients with various chemotherapy regimens (*P* = 0.083). Nevertheless, the OS (Fig. 4a, *P *= 0.006) and PFS (Fig. 4b, *P *= 0.03) of patients who received chemotherapy plus radiotherapy were significantly longer than those of patients who received chemotherapy alone. After treatment, there was no statistical difference in LMR levels among patients treated with various chemotherapy regimens (*P* = 0.148), however, the LMR of patients with chemotherapy plus radiotherapy was higher than that of patients with chemotherapy alone in both high and low LMR groups (Fig. 5, *P* = 0.017 and 0.037, respectively).

### Decreased LMR during follow-up was associated with disease recurrence

**Fig. 6:**
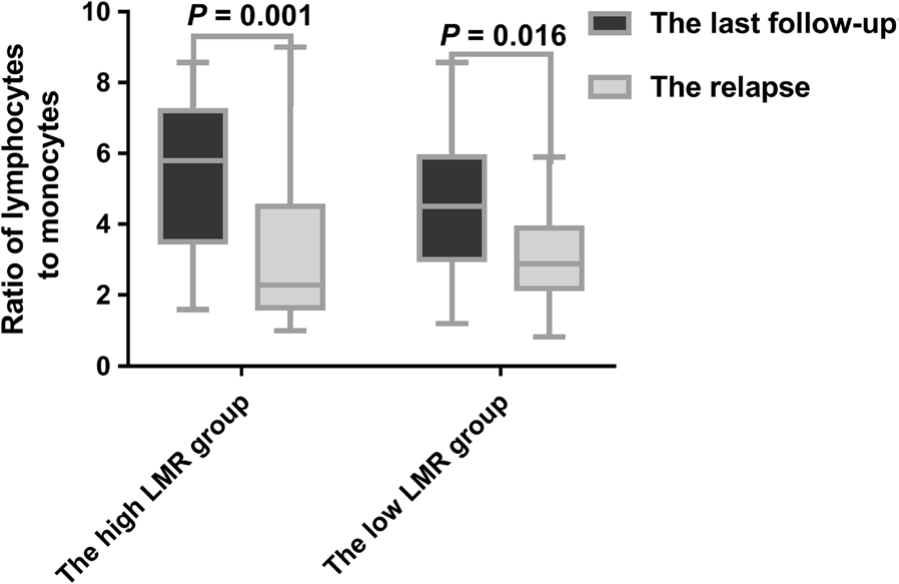
The reduction of LMR level during follow-up was associated with disease recurrence. Compared with the LMR level at the final follow-up, the LMR level at relapse was significantly lower in both high and low LMR groups (*P* value were 0.001 and 0.016, respectively)

The LMR was dynamically monitored at the follow-up of 3, 6, 9 and 12 months after treatment. The level of LMR increased over time, and eventually tended to a certain level in both groups. Nevertheless, compared with the LMR level at the last follow-up, the LMR level at relapse was significantly lower in both high and low LMR groups (Fig. 6, *P* = 0.001 and 0.016, respectively).

### Survival analysis and prognosis factors

Univariate analysis was shown in Table 2. ECOG PS > 2, local tumor invasiveness, B symptoms, ALC ≤ 1.5 × 10^9^/L, elevated serum LDH level, LMR ≤ 2.7, staging (III/IV), and chemotherapy or radiotherapy alone could be significantly associated with shorten OS and PFS. Clinical factors with statistically significance for OS and PFS (*P* < 0.05) were included in the multivariate analysis (Table 3). Due to overlap with several clinical factors, IPI and KPI were not included into the survival analysis. Multivariate analysis demonstrated that LMR was a poor prognostic factor for OS (*P *= 0.009) and PFS (*P* = 0.025).

**Table 2:** Univariate analysis of risk factors associated with OS and PFS

Variables	OS		PFS	
	HR (95% CI)	*P* value	HR (95% CI)	*P* value
Age (> 60 vs. ≤ 60)	1.80 (0.98–3.81)	0.065	1.04 (0.52–2.30)	0.205
Gender (male vs. female)	1.45 (1.07–1.92)	0.052	0.29 (0.13–0.86)	0.905
B symptoms (yes vs. no)	0.68 (0.24–1.63)	0.024	0.35 (0.12–1.98)	0.032
Lugano stage (I–II_2_ vs. III–IV)	2.28 (1.28–4.14)	0.001	1.52 (0.65–3.58)	0.018
LDH (elevated vs. normal)	1.31 (1.04–1.67)	0.015	0.67 (0.33–1.42)	0.327
Local tumor invasiveness (yes vs. no)	0.46 (0.22–0.96)	0.024	0.32 (0.15–0.66)	0.037
Treatment (chemotherapy or radiotherapy vs. both)	0.67 (0.33–1.42)	0.004	0.35 (0.12–2.30)	0.041
Absolute lymphocyte count (≤ 1.5 × 10^9^/L vs. > 1.5 × 10^9^/L)	1.56 (0.72–3.40)	0.002	1.24 (0.62–2.08)	0.006
Absolute monocyte count (≤ 0.5 × 10^9^/L vs. > 0.5 × 10^9^/L)	0.53 (0.21–1.37)	0.102	0.45 (0.13–2.49)	0.361
LMR (≤ 2.7 vs. > 2.7)	4.21 (1.53–11.56)	0.000	2.78 (1.15–6.76)	0.034

**Table 3:** Multivariate analysis of risk factors associated with OS and PFS

Variables	OS		PFS	
	HR (95% CI)	*P* value	HR (95% CI)	*P* value
B symptoms (yes vs. no)	1.52 (1.08–2.16)	0.011	1.31 (1.04–1.67)	0.056
Lugano stage (I–II_2_ vs. III–IV)	2.64 (1.45–5.09)	0.162	1.70 (0.59–4.88)	0.327
LDH (elevated vs. normal)	0.55 (0.16–2.41)	0.098	0.36 (0.11–1.20)	0.172
Local tumor invasiveness (yes vs. no)	0.60 (0.32–1.89)	0.048	0.52 (0.25–1.11)	0.074
Treatment (chemotherapy alone or radiotherapy alone vs. both)	1.09 (0.10–3.21)	0.041	0.71 (0.22–2.30)	0.109
Absolute lymphocyte count (≤ 1.5 × 10^9^/L vs. > 1.5 × 10^9^/L)	2.05 (0.34–5.80)	0.200	1.57 (1.06–2.33)	0.428
LMR (≤ 2.7 vs. > 2.7)	1.95 (0.75–4.09)	0.009	1.41 (0.34–4.80)	0.025

## Discussion

Two clinical prognostic models for ENKTL, IPI and KPI, are limited in terms of prognostic capability, especially in the era of asparaginase-based chemotherapy [16]. In this retrospective study, for the first time, we found that the low LMR before treatment and a further reduction of LMR after treatment were an independent prognostic factor of treatment response and prognosis in patients with ENKTL. More specifically, low LMR before treatment had the potential to predict poor early response to treatments, as well as shorter OS and PFS. A reduction of LMR level again was also associated with disease recurrence during follow-up in patients with ENKTL.

Previous studies already demonstrated the involvement and prognostic values of EBV infection in ENKTL [17]. More recently, increasing studies investigate the values of immune factors such as ALC, AMC, LMR, etc. As a matter of fact, serum EBV DNA-positive was closely associated with the decrease of LMR [18]. This was also in line with our findings that the lower LMR had the potential to predict the poor survival of ENKTL patients.

In the present study, LMRs were dynamically monitored at various time points (before, during, and after treatment), and the relationships between LMR and therapeutic response, and survival were analyzed, respectively. As reported in the literatures [19, 20] and our findings, LMR level at diagnosis was correlated with survival in patients with ENKTL. A previous study showed that the LMR/LDH was a better predictor than LMR in patients with NHL [21]. Here, it was found that LMR/LDH highly coincided with LMR in predicting the survival of patients with ENKTL. Additionally, we found that the patients with the high LMR at diagnosis had a higher CR rate after two or four courses of chemotherapy, which suggested that the high LMR level could be used as an indicator of good early response to treatments [22]. Furthermore, as reported in diffuse large B-cell lymphoma [23], this study found that the survival time of ENKTL patients with further increased LMR after treatment was significantly longer than that of patients with further decreased LMR in both high and low LMR groups, indicating that patients with increased LMR after treatment had faster immune function reconstruction, more likely to benefit from chemotherapy with better outcomes. By contrast, patients with lower LMR after treatment had poor prognosis, and may need more intensive chemotherapy or even stem cell transplantation. More importantly, we, for the first time, found the LMR level at relapse decreased significantly again during follow-up, suggesting that the reduction of LMR level may also be used as an indicator of risk for disease recurrence in patients with ENKTL.

ENKTL is a highly aggressive lymphoma. Fortunately, asparaginase-based regimens significantly improve the prognosis [24]. The rationale behind using asparaginase agents as an anti-cancer treatment is that normal lymphocytes have the asparagine synthetase enzyme and can promote the biosynthesis of their own asparagine, whereas this does not occur in lymphoma cells [25]. Thus, those agents showed more cytotoxicity on malignant lymphocytes than normal cells, leading to a decrease in lymphocytes [26–28], which probably explained why the LMR level in high LMR group decreased significantly when treatment response was achieved in the present study, and the incidence of infection increased during the treatment of asparaginase agents [27, 28]. Furthermore, the OS and PFS in patients treated with chemotherapy plus radiotherapy were superior to that of patients with chemotherapy alone. Interestingly, the LMR levels after treatment in patients treated with chemotherapy plus radiotherapy were significantly higher than those of patients treated with chemotherapy alone, consistent with previous report in solid tumors [29]. This phenomenon may be associated with that radiation-induced necrosis of tumor tissue, as a tumor vaccine, induces lymphocyte proliferation [30–32].

However, there are still some limitations in this study e.g., this was a single-center and retrospective study. Thus, it is necessary to conduct a multi-center and prospective study, and to expand the sample size to verify these results in the future.

In conclusion, this study, for the first time, retrospectively analyzed the correlation between the dynamic changes of LMR at various time points and the treatment responses, prognosis, and recurrence in ENKTL patients. We found that low LMR at diagnosis was a simple and effective indicator of poor response to treatment and poor prognosis. Additionally, further reduction of LMR during follow-up was associated with disease recurrence.

## Data Availability

Not applicable.

## Abbreviations

ENKTL: extranodal NK/T cell lymphoma
CR: complete remission
LMR: lymphocyte to monocyte ratio
PR: partial remission
OS: overall survival
PFS: progression-free survival
ECOG: Eastern Cooperative Oncology Group
PS: performance status
SD: stationary disease
PD: progressive disease
LDH: lactate dehydrogenase
β2 M: β2 microglobulin
KPI: Korean Prognosis Index
IPI: International Prognostic Index
