# Correction to: The predictive value of dynamic monitoring of peripheral blood lymphocyte to monocyte ratio in patients with extranodal NK/T cell lymphoma

**DOI:** 10.1186/s12935-020-1102-9

**Published:** 2020-01-16

**Authors:** Shengnan Zhang, Mengjuan Li, Fangfang Yuan, Lin Chen, Ruihua Mi, Xudong Wei, Yongping Song, Qingsong Yin

**Affiliations:** Department of Hematopathy, Henan Institute of Hematology, Cancer Hospital Affiliated to Zhengzhou University, Zhengzhou, 450000 Henan China

## Correction to: Cancer Cell Int (2019) 19:272 10.1186/s12935-019-0993-9

After publication of our article [1] it was highlighted by the authors that the type of this article was primary research, instead of review.

## References

[1] Zhang S, Li M, Yuan F, Chen L, Mi R, Wei X, Song Y, Yin Q (2019). The predictive value of dynamic monitoring of peripheral blood lymphocyte to monocyte ratio in patients with extranodal NK/T cell lymphoma. Cancer Cell Int.

